# A three-dimensional particle finite element model for simulating soil flow with elastoplasticity

**DOI:** 10.1007/s11440-022-01618-1

**Published:** 2022-06-28

**Authors:** Liang Wang, Xue Zhang, Qinghua Lei, Stelios Panayides, Stefano Tinti

**Affiliations:** 1 Department of Earth Sciences, ETH Zürich, Zürich, Switzerland; 2 Dipartimento di Fisica e Astronomia “Augusto Righi” (DIFA), Settore di Geofisica, Università di Bologna, Bologna, Italy; 3 Department of Civil Engineering and Industrial Design, School of Engineering, University of Liverpool, Liverpool, UK; 4 Subsea 7, Sutton, UK

**Keywords:** Large deformation, Second-order cone programming, Slope stability, Soil flow, PFEM

## Abstract

Soil flow is involved in many earth surface processes such as debris flows and landslides. It is a very challenging task to model this large deformational phenomenon because of the extreme change in material configurations and properties when soil flows. Most of the existing models require a two-dimensional (2D) simplification of actual systems, which are however three-dimensional (3D). To overcome this issue, we develop a novel 3D particle finite element method (PFEM) for direct simulation of complex soil flows in 3D space. Our PFEM model implemented in a fully implicit solution framework based on a generalised Hellinger–Reissner variational principle permits the use of a large time step without compromising the numerical stability. A mixed quadratic-linear element is used to avoid volumetric locking issues and ensure computational accuracy. The correctness and robustness of our 3D PFEM formulation for modelling large deformational soil flow problems are demonstrated by a series of benchmarks against analytical or independent numerical solutions. Our model can serve as an effective tool to support the assessment of catastrophic soil slope failures and subsequent runout behaviours.

## Introduction

In the framework of the classical Lagrangian finite element method (FEM), computational meshes move with material configurations they are attached to, allowing to conveniently track material motion and handle history-dependent materials. Nevertheless, severe mesh distortion may occur if the material undergoes large deformation, which leads to a significant reduction in the accuracy of numerical solutions and even non-convergence of numerical simulations. Thus, over the past decades, extensive efforts have been devoted to the development of advanced numerical approaches to simulate large deformation problems in geotechnical engineering. An example of mesh-based methods for tackling this issue is the well-known arbitrary Lagrangian Eulerian (ALE) method [41, 50]. The ALE method enables mesh points to move according to designed trajectories that may not coincide with either the material or spatial configuration. In such a way, it does eliminate mesh distortion although the design of appropriate trajectories is non-trivial for problems involving complex geometrical changes. A pure particle-based method to alleviate mesh distortion is the so-called smooth particle hydrodynamics (SPH) method [6, 45, 75], which uses particles to represent computational domains and construct interpolation functions without mesh discretisation. Thus, it can handle problems with no limitation on the deformation extent, even for the extreme scenarios with severe free-surface evolution such as soil splashing during landslides**.** A more detailed review of the recent development and application of SPH in geomechanics can be found in [7]. Some hybrid methods using both particles and finite element meshes have also been developed, an example of which is the material point method (MPM) [15, 38, 48, 51, 56], originally named particle-in-cell method [24]. In this method, the material is represented as a cloud of Lagrangian particles, called materials points, where the information of physical properties and state variables are stored. The governing equations are resolved on the Eulerian background meshes. At each incremental time step, state variables and physical properties are mapped between particles and background meshes. Another hybrid method attracting an increasing attention is the particle finite element method (PFEM). Originally invented for solving fluid mechanics problems [27, 43], the PFEM has been further developed and applied to study a wide range of challenging problems across different disciplines. Below, we give a brief summary of its main concept as well as the current state in its development and application. For a more thorough review of the PFEM, the reader can refer to [13].

The fundamental idea underlying the PFEM is its treatment of mesh nodes as particles. The PFEM model solves governing equations in a time-marching fashion based on the conventional Lagrangian FEM framework with finite element meshes. Mesh connectivity, however, is erased at the end of each time step leaving mesh nodes treated as free particles. Before moving to the next time step, a new computational domain is identified according to the location of particles, followed by the construction of a new mesh of satisfactory quality. By doing so, the PFEM inherits both the solid mathematical foundation of the traditional FEM and the flexibility of particle-based approaches for handling large deformations. To date, the PFEM and its variants have been extensively developed and applied to study various geomechanical and geotechnical problems including (but not limited to) soil-structure interactions [3, 10, 25, 40, 46, 66], granular flows [9, 14, 28, 35, 64, 73], progressive soil slope failures [57, 63], debris flows [21], cliff erosion [39], landslide events [65, 70] and landslide-induced waves [47, 69]. Despite its substantial development, most of the existing PFEM models for geomechanical/geotechnical engineering applications can only deal with two-dimensional (2D) problems, whereas very limited work has been conducted for three-dimensional (3D) modelling, reviewed as follows. A 3D PFEM was developed in the framework of computational fluid dynamics to solve the Navier–Stokes equations and further applied to simulate debris flows and landslide problems [17, 21], where soils are modelled as non-Newtonian fluid. A variant of the 3D PFEM model was developed for elastoplastic analysis of soils [72], where the strain field is smoothed so that first-order elements (i.e. four-node tetrahedron elements) can be used without encountering volumetric locking issues. Additionally, the 3D PFEM model developed in [72] has been parallelised with GPUs to improve its computational efficiency given that the employed explicit FEM formulation requires very small time steps.

In this paper, a new 3D PFEM model is developed for simulating large deformation soil flows based on the generalised Hellinger–Reissner variational principle [69] and an elastoplastic constitutive law. An implicit time integration scheme is adopted enabling the use of a relatively large time step which is of great advantage in solving geomechanical/geotechnical problems usually of quasi-static or low-to-medium dynamic nature. A mixed quadratic/linear tetrahedral element is used with a quadratic interpolation for displacements and inertia forces and a linear interpolation for stresses. The problem of standard second-order cone programming (SOCP) is resolved efficiently using the interior-point method [1, 31, 60]. The developed model inherits the advantages of SOCP-FEM: (i) handling the singularities of constitutive models (e.g. Bingham, Mohr–Coulomb and Drucker Prager models) [4, 34]; (ii) the extension from single-surface plasticity to multi-surface plasticity [34]; (iii) the efficiency of high-performance SOCP solvers in large-scale problems [22]. For verification, a series of benchmark tests are designed and analysed using the proposed 3D PFEM model, with the simulation results compared against analytical solutions, experimental data, and independent numerical simulations. The computational cost of our model is also presented to show its efficiency.

The rest of the paper is structured as follows. Section 2 summarises the governing equations for elastoplastic dynamic analysis of soils, the time domain discretisation, the corresponding variational principle and the spatial domain discretisation. Section 3 describes the reformulation and computation of the optimisation problem. The computational procedures of the 3D PFEM are given in Sect. 4. Numerical examples are shown in Sect. 5 and conclusions are drawn in Sect. 6.

## Mathematical formulation and numerical methods

### Governing equations

We consider a 3D domain *V*, delimited by a surface *S*. The set of equations governing the dynamic elastoplastic behaviour of the material is given below.

- Equilibrium equation:
  1 $$\varvec{\nabla}^{{\text{T}}} {{\varvec{\upsigma}}} + {\mathbf{b}} = \rho {\dot{\mathbf{v}}}{\text{, in }}V$$
  where **σ** = (*σ*_*xx*_, *σ*_*yy*_, *σ*_*zz*_, *σ*_*xy*_, *σ*_*xz*_, *σ*_*yz*_)^T^ is the stress, **v** = (*v*_*x*_, *v*_*y*_, *v*_*z*_)^T^ is the velocity, **b** is the body force, *ρ* is the density, ${\dot{\mathbf{v}}}$ is the time derivative of **v**, and $\varvec{\nabla}^{{\text{T}}}$ is the differential operator defined as
  2 $$\varvec{\nabla}^{{\text{T}}} = \left( {\begin{array}{*{20}l} {\frac{\partial }{\partial x}}   & 0   & 0   & 0   & {\frac{\partial }{\partial z}}   & {\frac{\partial }{\partial y}}   \\ 0   & {\frac{\partial }{\partial y}}   & 0   & {\frac{\partial }{\partial z}}   & 0   & {\frac{\partial }{\partial x}}   \\ 0   & 0   & {\frac{\partial }{\partial z}}   & {\frac{\partial }{\partial y}}   & {\frac{\partial }{\partial x}}   & 0   \\ \end{array} } \right)^{{\text{T}}}$$
- Displacement–strain relation:
  3 $${{\varvec{\upvarepsilon}}} = {\varvec{\nabla}}{\mathbf{u}}$$
  where **ɛ** = (*ɛ*_*xx*_, *ɛ*_*yy*_, *ɛ*_*zz*_, *2ɛ*_*xy*_, *2ɛ*_*xz*_, *2ɛ*_*yz*_)^T^ is the strain and **u** = (*u*_*x*_, *u*_*y*_, *u*_*z*_)^T^ is the displacement.
- Boundary conditions:
  4 $${\mathbf{N}\varvec{\upsigma}} = {\mathbf{t}}\;\quad {\text{on}}\;\quad S_{\text{t}}$$
  5 $${\mathbf{u}} = {\mathbf{u}}^{{\text{p}}} \;\quad{\text{on}}\;\quad S_{\text{u}}$$
  where **N** is the unit normal vector, **t** is the traction, **u**^p^ is the prescribed displacement, and *S*_t_ and *S*_u_ are the surfaces where traction and displacement are imposed, respectively.
- Constitutive equations:
  6 $$F({{\varvec{\upsigma}}}) \le 0$$
  7 $${{\varvec{\upvarepsilon}}} = {{\varvec{\upvarepsilon}}}^{{\text{e}}} + {{\varvec{\upvarepsilon}}}^{{\text{p}}} , \, {{\varvec{\upvarepsilon}}}^{{\text{e}}} = {\mathbb{C}}{{\varvec{\upsigma}}}, \, {{\varvec{\upvarepsilon}}}^{{\text{p}}} = \lambda \varvec{\nabla} G({{\varvec{\upsigma}}})$$

where *F* is the yield function, **ɛ**^e^ and **ɛ**^p^ are the elastic and plastic strains, respectively, ${\mathbb{C}}$ is the elastic compliance matrix, *λ* is the plastic multiplier, and *G* is the plastic potential. The associated flow rule assumes *G* = *F*, and the so-called complementary condition for the incremental form of Eqs. (6) and (7) can be written as follows

8 $$\left\{ {\begin{array}{*{20}c} {F({{\varvec{\upsigma}}}) \le 0}\;\;\;\;\;\;\;\;\;\;\quad\quad\quad\quad \\ {\Delta {{\varvec{\upvarepsilon}}} = \mathbb{C}\Delta {{\varvec{\upsigma}}} + \Delta \lambda {\varvec{\nabla} }F({{\varvec{\upsigma}}})} \\ {\Delta \lambda F({{\varvec{\upsigma}}}) = 0, \, \Delta \lambda \ge 0}\;\;\;\;\; \\ \end{array} } \right.$$

### Time discretisation

In the framework of the *θ*-method [58], the stress and velocity evolution may be described in a time marching fashion as

9 $${{\varvec{\upsigma}}} = \theta_{1} {{\varvec{\upsigma}}}_{n + 1} + (1 - \theta_{1} ){{\varvec{\upsigma}}}_{n}$$

10 $${\mathbf{v}} = {\dot{\mathbf{u}}} = \frac{{{\mathbf{u}}_{n + 1} - {\mathbf{u}}_{n} }}{\Delta t} = \frac{{\Delta {\mathbf{u}}}}{\Delta t} = \theta_{2} {\mathbf{v}}_{n + 1} + (1 - \theta_{2} ){\mathbf{v}}_{n}$$

with Eq. (1) also discretised as

11 $$\varvec{\nabla}^{{\text{T}}} [\theta_{1} {{\varvec{\upsigma}}}_{n + 1} + (1 - \theta_{1} ){{\varvec{\upsigma}}}_{n} ] + {\mathbf{b}} = \rho \frac{{{\mathbf{v}}_{n + 1} - {\mathbf{v}}_{n} }}{\Delta t}$$

where the subscripts *n* and *n* + 1 denote the known and unknown states, Δ*t* is the time step, and 0 ≤ *θ*_1_, *θ*_2_ ≤ 1 are numerical parameters. By introducing a new variable **r**, Eq. (11) is rearranged as

12 $$\varvec{\nabla}^{{\text{T}}} {{\varvec{\upsigma}}}_{n + 1} + \frac{{(1 - \theta_{1} )}}{{\theta_{1} }}\varvec{\nabla}^{{\text{T}}} {{\varvec{\upsigma}}}_{n} + {\tilde{\mathbf{b}}} = {\mathbf{r}}_{n + 1}$$

with traction boundary conditions

13 $${\mathbf{N }{\varvec{\upsigma}}}_{n + 1} = {\tilde{\mathbf{t}}}$$

in which

14 $${\tilde{\mathbf{b}}} = \frac{1}{{\theta_{1} }}{\mathbf{b}} + \tilde{\rho }\frac{{{\mathbf{v}}_{n} }}{\Delta t},\tilde{\rho } = \frac{\rho }{{\theta_{1} \theta_{2} }},{\mathbf{r}}_{n + 1} = \tilde{\rho }\frac{{\Delta {\mathbf{u}}}}{{\Delta t^{2} }},{\tilde{\mathbf{t}}} = \frac{1}{{\theta_{1} }}{\mathbf{t}} - \frac{{1 - \theta_{1} }}{{\theta_{1} }}{\mathbf{N}}{\varvec{\upsigma}}_{n}$$

The update of velocity is based on Eq. (10), such that the new velocity is given as

15 $${\mathbf{v}}_{n + 1} = \frac{1}{{\theta_{2} }}\left[ {\frac{{\Delta {\mathbf{u}}}}{\Delta t} - (1 - \theta_{2} ){\mathbf{v}}_{n} } \right]$$

The time integration scheme adopted in our model is unconditionally stable for *θ*_1_ ≥ 0.5 and *θ*_2_ ≥ 0.5. Despite its simplicity, the *θ*-method fits the mixed variational principle (to be introduced in Sect. 2.3) very well. Notably, it has also been successfully adopted to simulate challenging dynamic problems of saturated soils in a similar framework [55].

### Mixed variational principle

A mixed variational principle has been previously established in [69] for dynamic elastoplastic problems which read

16 $$ \begin{aligned}  \mathop {\min }\limits_{{\Delta {\mathbf{u}}}} \, \mathop {{\text{max}}}\limits_{{({{\varvec{\upsigma}}}\user2{, }{\mathbf{r}}{)}_{n + 1} }} \,& - \frac{1}{2}\int_{V} {\Delta {{\varvec{\upsigma}}}^{{\text{T}}} \mathbb{C}\Delta {{\varvec{\upsigma}}}dV} + \int_{V} {{{\varvec{\upsigma}}}_{n + 1}^{{\text{T}}} \varvec{\nabla} (\Delta {\mathbf{u}})dV} \\&+ \frac{{(1 - \theta_{1} )}}{{\theta_{1} }}\int_{V} {{{\varvec{\upsigma}}}_{n}^{{\text{T}}} \varvec{\nabla} (\Delta {\mathbf{u}})dV} \, - \int_{V} {{\tilde{\mathbf{b}}}^{{\text{T}}} \Delta {\mathbf{u}}dV}\, \\& \quad - \int_{S} {{\tilde{\mathbf{t}}}^{{\text{T}}} \Delta {\mathbf{u}}dS} + \int_{V} {{\mathbf{r}}_{n + 1}^{{\text{T}}} \varvec{\nabla} (\Delta {\mathbf{u}})dV}\\&\quad\quad\,\, - \frac{{\Delta t^{2} }}{2}\int_{V} {{\mathbf{r}}_{n + 1}^{{\text{T}}} \tilde{\rho }^{ - 1} {\mathbf{r}}_{n + 1} dV}   \\ {\text{subject to }} &\quad\quad\quad \quad\quad F({{\varvec{\upsigma}}}_{n + 1} ) \le 0   \\ \end{aligned}$$

According to [69], the Lagrangian of the above min–max optimisation problem is

17 $$\begin{gathered} \mathcal{L}\left( {\Delta {\mathbf{u}},{{\varvec{\upsigma}}}_{{n{ + 1}}} ,{\mathbf{r}}_{{n{ + 1}}} ,\Delta \lambda } \right) = \int_{V} {{{\varvec{\upsigma}}}_{{n{ + 1}}}^{{\text{T}}} \varvec{\nabla}^{{\text{T}}} (\Delta {\mathbf{u}}){\text{d}}V} { + \int_{V} {\frac{{1 - \theta_{1} }}{{\theta_{1} }}{{\varvec{\upsigma}}}_{n}^{{\text{T}}} \varvec{\nabla} (\Delta {\mathbf{u}}){\text{d}}V} - \int_{V} {{\tilde{\mathbf{b}}}^{{\text{T}}} \Delta {\mathbf{u}}{\text{d}}V} - \int_{{S_{{\text{t}}} }} {{\tilde{\mathbf{t}}}^{{\text{T}}} \Delta {\mathbf{u}}{\text{d}}S} }   \\ \;\;\; - \frac{1}{2}\int_{V} \Delta {{\varvec{\upsigma}}}^{{\text{T}}} \mathbb{C}\Delta {{\varvec{\upsigma}}}dV - \frac{1}{2}\int_{V} {{\mathbf{r}}_{{n{ + 1}}}^{{\text{T}}} \frac{{\Delta t^{2} }}{{\tilde{\rho }}}{\mathbf{r}}_{{n{ + 1}}}^{{}} {\text{d}}V} + \int_{V} {{\mathbf{r}}_{{n{ + 1}}}^{{\text{T}}} \Delta {\mathbf{u}}{\text{d}}V} - \int_{V} {\Delta \lambda F({{\varvec{\upsigma}}}_{{n{ + 1}}} )} {\text{d}}V   \\ \end{gathered}$$

The solution to problem (16) is a saddle point of its functional satisfying the associated Karush–Kuhn–Tucker (KKT) conditions which are.

- Stationarity:
  18 $$\frac{{\partial \mathcal{L}}}{{\partial \Delta {\mathbf{u}}}} = \left\{ {\begin{array}{*{20}l} {\varvec{\nabla}^{{\text{T}}} {{\varvec{\upsigma}}}_{{n{ + 1}}}^{{}} + \frac{{1 - \theta_{1} }}{{\theta_{1} }}\varvec{\nabla}^{{\text{T}}} {{\varvec{\upsigma}}}_{n}^{{}} + {\tilde{\mathbf{b}}} - {\mathbf{r}}_{{n{ + 1}}}^{{}} = {\mathbf{0}}\quad {\text{in}}\;V}   \\ {{\mathbf{N}}^{{\text{T}}} ({{\varvec{\upsigma}}}_{{n{ + 1}}} + \frac{{1 - \theta_{1} }}{{\theta_{1} }}{{\varvec{\upsigma}}}_{n}^{{}} ) = {\tilde{\mathbf{t}}}\quad {\text{on}}\;S_{{\text{t}}} }   \\ \end{array} } \right.$$
  19 $$\frac{{\partial \mathcal{L}}}{{\partial {{\varvec{\upsigma}}}_{{n{ + 1}}} }} = \varvec{\nabla} (\Delta {\mathbf{u}}) - \mathbb{C}({{\varvec{\upsigma}}}_{{n{ + 1}}} - {{\varvec{\upsigma}}}_{n} ) - \Delta \lambda \frac{\partial F}{{\partial {\varvec{\sigma}}_{{n{ + }1}} }} = {\mathbf{0}}\quad {\text{in}}\;V$$
  20 $$\frac{{\partial \mathcal{L}}}{{\partial {\mathbf{r}}_{{n{ + 1}}} }} = \Delta t^{2} \tilde{\rho }^{ - 1} {\mathbf{r}}_{{n{ + 1}}}^{{}} - \Delta {\mathbf{u}} = {\mathbf{0}}\quad {\text{in}}\;V$$
- Complementary slackness:
  21 $$\Delta \lambda F({{\varvec{\upsigma}}}_{{n{ + 1}}}^{{}} ) = 0$$
- Primal feasibility:
  22 $$F({{\varvec{\upsigma}}}_{{n{ + 1}}}^{{}} ) \le 0$$
- Dual feasibility:
  23 $$\lambda \ge 0$$

One can see that the KKT conditions (18)–(23) are exactly the governing equations (1)–(8) after time discretisation, which verifies the variational principle (16).

### Spatial domain discretisation

**Fig. 1:**
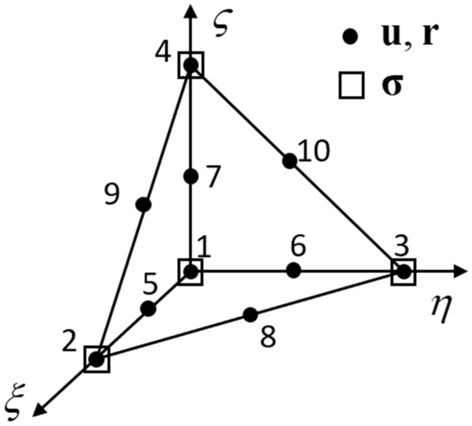
Mixed isoparametric tetrahedral element utilised in the simulation: Linear interpolation nodes including 1 (0,0,0), 2 (1,0,0), 3 (0,1,0), and 4 (0,0,1); Quadratic interpolation nodes consisting of the four linear interpolation nodes 1–4 and six middle nodes 5 (0.5,0,0), 6 (0,0.5,0), 7 (0,0,0.5), 8 (0.5,0.5,0), 9 (0.5,0,0.5), and 10 (0,0.5,0.5). Here, the values inside the bracket indicate the coordinates of the corresponding node in the normalised coordinate system (*ξ*, *η*, *ς*)

We describe the spatial domain discretisation of the min–max problem (16) below. A mixed isoparametric tetrahedral element shown in Fig. 1 is adopted. In this element, quadratic interpolation is used for the fields of displacement **u**, and inertia force **r**, whilst linear interpolation is used for the field of stress **σ**. Owing to the use of non-equal interpolations for the stress and displacement fields, this quadratic/linear mixed element overcomes volumetric locking for incompressible/near-incompressible problems.

Using the standard finite element notation, the fields are approximated as

24 $${{\varvec{\sigma}}} \approx {\mathbf{N}}_{\sigma } {\hat{\varvec{\sigma }}},\;{\mathbf{u}} \approx {\mathbf{N}}_{u} {\hat{\mathbf{u}}},\;{\mathbf{r}} \approx {\mathbf{N}}_{r} {\hat{\mathbf{r}}}$$

where ${\hat{\varvec{\sigma }}}$, ${\hat{\mathbf{u}}}$ and ${\hat{\mathbf{r}}}$ are the stresses, displacements and dynamic forces at the nodes, while **N**_*σ*_, **N**_*u*_ and **N**_*r*_ are the matrices of the corresponding shape functions. The strains are

25 $${{\varvec{\upvarepsilon}}} = \varvec{\nabla} {\mathbf{u}} \approx {\mathbf{B}}_{u} {\hat{\mathbf{u}}}\quad {\text{with}}\;{\mathbf{B}}_{u} = \varvec{\nabla} {\mathbf{N}}_{u}$$

Substituting (24) into (16), the discretised min–max problem is written as

26 $$\begin{gathered} \mathop {\min }\limits_{{\Delta {\hat{\mathbf{u}}}}} \, \mathop {{\text{max}}}\limits_{{({\hat{\varvec{\sigma }}}\user2{, }{\hat{\mathbf{r}}}{)}_{{n{ + 1}}} }} \, - \frac{1}{2}\Delta {\widehat{\varvec{\sigma }}}^{{\text{T}}} {\mathbf{C}}\Delta {\widehat{\varvec{\sigma }}} - \frac{1}{2}\Delta t^{2} {\hat{\mathbf{r}}}_{{n{ + 1}}}^{{\text{T}}} {\mathbf{D\hat{r}}}_{{n{ + 1}}} + {\widehat{\varvec{\sigma }}}_{{n{ + 1}}}^{{\text{T}}} {\mathbf{B}}\Delta {\hat{\mathbf{u}}} - {\tilde{\mathbf{f}}}^{{\text{T}}} \Delta {\hat{\mathbf{u}}} + {\hat{\mathbf{r}}}_{{n{ + 1}}}^{{\text{T}}} {\mathbf{A}}\Delta {\hat{\mathbf{u}}}   \\ {\text{subject to }}\quad F({\widehat{\varvec{\sigma }}}_{{n{ + 1}}} ) \le 0   \\ \end{gathered}$$

where

27 $${\mathbf{B}} = \int_{V} {\mathbf{N}}_{\sigma }^{{\text{T}}} {\mathbf{B}}_{u} dV$$

28 $${\mathbf{C}} = \int_{V} {\mathbf{N}}_{\sigma }^{{\text{T}}} \mathbb{C}{\mathbf{N}}_{\sigma } dV$$

29 $${\mathbf{D}} = \int_{V} {\mathbf{N}}_{r}^{{\text{T}}} \tilde{\rho }^{ - 1} {\mathbf{N}}_{r} dV$$

30 $${\mathbf{A}} = \int_{V} {\mathbf{N}}_{r}^{{\text{T}}} {\mathbf{N}}_{u} dV$$

31 $${\tilde{\mathbf{f}}} = \int_{V} {\mathbf{N}}_{u}^{{\text{T}}} {\tilde{\mathbf{b}}}dV + \int_{S} {\mathbf{N}}_{u}^{{\text{T}}} {\tilde{\mathbf{t}}}dS - \frac{{(1 - \theta_{1} )}}{{\theta_{1} }}{\mathbf{B}}^{{\text{T}}} {\hat{\varvec{\sigma }}}_{n}$$

## Reformulation and computation of the optimisation problem

In this section, the min–max problem (26) is reformulated as a standard second-order cone programming (SOCP) problem

32 $$\begin{gathered} \mathop {\min }\limits_{{\mathbf{x}}} \,\quad \quad \quad {\mathbf{p}}^{{\text{T}}} {\mathbf{x}}   \\ {\text{subject to }}\quad {\mathbf{Lx}}{ = }{\mathbf{y}}   \\ \,\quad \quad \quad \quad \quad {\mathbf{x}} \in \aleph   \\ \end{gathered}$$

where ${\mathbf{x}} = (x_{1} ,x_{2} ,...,x_{n} )^{{\text{T}}}$ consists of the field variables, **L** and **y** are the known matrix and vector for the linear equality, **p** is the vector of coefficients for the objective function, and $\aleph$ is the tensor product of second-order cones such that $\aleph = \aleph_{1} \times \cdots \times \aleph_{l}$. The second-order cones can be in the type of:

• Quadratic cone

33 $$\aleph_{q}^{n} = \left\{ {{\mathbf{x}} \in \Re^{n} : \, x_{1} \ge \sqrt {x_{2}^{2} + \cdots + x_{n}^{2} } } \right\}$$

• Rotated quadratic cone

34 $$\aleph_{r}^{n} = \left\{ {{\mathbf{x}} \in \Re^{n} :{ 2}x_{1} x_{2} \ge \sum\limits_{j = 3}^{n} {x_{j}^{2} , \, x_{1,2} \ge 0} } \right\}$$

To this end, the minimisation part of (26) is first resolved analytically, leading to

35 $$\begin{gathered} \quad \quad \quad\mathop {\max }\limits_{{({\hat{\varvec{\sigma }}},\widehat{{\mathbf{r}}})_{{n + 1}} }} \quad \; - \frac{1}{2}\Delta {\widehat{\varvec{\sigma }}}^{{\text{T}}} {\mathbf{C}}\Delta {\widehat{\varvec{\sigma }}} - \frac{{\Delta t^{2} }}{2}\widehat{{\mathbf{r}}}_{{n + 1}}^{{\text{T}}} \, {\mathbf{D}}\widehat{{\mathbf{r}}}_{{n + 1}}   \\ {\text{subject}}\;{\text{to }} \quad{\mathbf{B}}^{{\text{T}}} {\widehat{\varvec{\sigma }}}_{{n + 1}} + \mathbf{A}^{{\text{T}}} \widehat{{\mathbf{r}}}_{{n + 1}} = \widetilde{{\mathbf{f}}}   \\ \quad \quad \quad \quad F({\widehat{\varvec{\sigma }}}_{{n + 1}} ) \le 0   \\ \end{gathered}$$

Following [64], the above maximisation problem is equivalent to the minimisation problem below

36 $$\begin{gathered} \mathop {\quad \quad\min }\limits_{{({\hat{\varvec{\sigma }}},\hat{\mathbf{r}}{)}_{{n{ + 1}}},s_{1} ,s_{2}}} \,\quad s_{1} + s_{2}   \\ {\text{subject to }}\quad{\mathbf{B}}^{{\text{T}}} {\hat{\varvec{\sigma }}}_{{n{ + 1}}} + {\mathbf{A}}^{{\text{T}}} {\hat{\mathbf{r}}}_{{n{ + 1}}} = {\tilde{\mathbf{f}}}   \\ \quad \quad \quad \quad \;\;\;\frac{1}{2} \Delta \widehat{\user2{\sigma }}^{{\text{T}}} {\mathbf{C}}\Delta \widehat{\varvec{\sigma }} \le s_{1}   \\ \quad \quad \quad \quad \;\;\;\frac{{\Delta t^{2} }}{2}{\hat{\mathbf{r}}}_{{n{ + 1}}}^{{\text{T}}} \; {\mathbf{D}}\,{\hat{\mathbf{r}}}_{{n{ + 1}}} \le s_{2}   \\ \, F({\hat{\varvec{\sigma }}}_{{n{ + 1}}} ) \le 0   \\ \end{gathered}$$

where $s_{1}$ and $s_{2}$ are unknown positive scalars. By introducing ${{\varvec{\upxi}}}_{\sigma }^{{}} = \sqrt {\mathbf{C}} \Delta {\hat{\mathbf{\sigma }}}$ and ${{\varvec{\upxi}}}_{r}^{{}} = \Delta t\sqrt {\mathbf{D}} {\hat{\mathbf{r}}}_{{n{ + 1}}}$, the above problem is further expressed as

37 $$\begin{gathered} \mathop {\min }\limits_{{({\hat{\varvec{\sigma }}},{\hat{\mathbf{r}}}{)}_{{n{ + 1}}} ,s_{1} ,s_{2} ,{{\varvec{\upxi}}}_{\sigma }^{{}} ,{{\varvec{\upxi}}}_{r}^{{}} }} \, s_{1} + s_{2}   \\ {\text{subject to }}\quad{\mathbf{B}}^{{\text{T}}} {\hat{\varvec{\sigma }}}_{{n{ + 1}}} + {\mathbf{A}}^{{\text{T}}} {\hat{\mathbf{r}}}_{{n{ + 1}}} = {\tilde{\mathbf{f}}}   \\ \quad \quad \quad \quad \;\;\;\quad\quad{{\varvec{\upxi}}}_{\sigma }^{{}} = \sqrt {\mathbf{C}} \Delta {\hat{\varvec{\sigma }}};\quad \;\;\;\underline{{{{\varvec{\upxi}}}_{\sigma }^{{\text{T}}} {{\varvec{\upxi}}}_{\sigma }^{{}} \le 2s_{1} }}   \\\quad\quad \quad \quad \quad \quad \;\;\;{{\varvec{\upxi}}}_{r}^{{}} = \Delta t\sqrt {\mathbf{D}} {\hat{\mathbf{r}}}_{{n{ + 1}}} ;\;\;\;\underline{{{{\varvec{\upxi}}}_{r}^{{\text{T}}} {{\varvec{\upxi}}}_{r}^{{}} \le 2s_{2} }}   \\ \, F({\hat{\varvec{\sigma }}}_{{n{ + 1}}} ) \le 0   \\ \end{gathered}$$

where the underlined terms are rotated quadratic cones (i.e. (34)).

Additionally, the yield criterion in (37) has to be reformulated as cones. The Drucker-Prager model is adopted here with the yield function given as

38 $$F = \alpha I_{1} + \sqrt {J_{2} } - k \le 0$$

where $I_{1} = \sigma_{x} + \sigma_{y} + \sigma_{z}$ and $J_{2} = \frac{1}{6}[(\sigma_{x} - \sigma_{y} )^{2} + (\sigma_{y} - \sigma_{z} )^{2} + (\sigma_{z} - \sigma_{x} )^{2} ] + \sigma_{xy}^{2} + \sigma_{yz}^{2} + \sigma_{xz}^{2}$ are the first invariant of the total stress and the second invariant of the deviatoric stress, respectively. We assume that the Drucker-Prager yield surface middle circumscribes the Mohr–Coulomb yield surface. The material parameters α and *k* can then be calculated by

39 $$\alpha = \frac{2\sin \varphi }{{\sqrt 3 (3 + \sin \varphi )}}\;,k = \frac{6c\cos \varphi }{{\sqrt 3 (3 + \sin \varphi )}}$$

where *φ* and *c* are the friction angle and cohesion of the Mohr–Coulomb yield function, respectively.

To cast the yield criterion as a standard cone, a new set of variables ${{\varvec{\uprho}}} = (\rho_{1} ,\rho_{2} ,\rho_{3} ,\rho_{4} ,\rho_{5} ,\rho_{6} ,\rho_{7} )^{{\text{T}}}$ is introduced

40 $${{\varvec{\uprho}}} = {\mathbf{H}}{{\varvec{\upsigma}}} + {\mathbf{d}}$$

where

41 $${\mathbf{H}} = \left[ {\begin{array}{*{20}c} { - \alpha } & { - \alpha } & { - \alpha } & {\begin{array}{*{20}c} 0 & 0 & 0 \\ \end{array} } \\ {1/\sqrt 6 } & {-1/\sqrt 6 } & 0 & {\begin{array}{*{20}c} 0 & 0 & 0 \\ \end{array} } \\ 0 & {1/\sqrt 6 } & { - 1/\sqrt 6 } & {\begin{array}{*{20}c} 0 & 0 & 0 \\ \end{array} } \\ {\begin{array}{*{20}c} { - 1/\sqrt 6 } \\ 0 \\ {\begin{array}{*{20}c} 0 \\ 0 \\ \end{array} } \\ \end{array} } & {\begin{array}{*{20}c} 0 \\ 0 \\ {\begin{array}{*{20}c} 0 \\ 0 \\ \end{array} } \\ \end{array} } & {\begin{array}{*{20}c} {1/\sqrt 6 } \\ 0 \\ {\begin{array}{*{20}c} 0 \\ 0 \\ \end{array} } \\ \end{array} } & {\begin{array}{*{20}c} {\begin{array}{*{20}c} 0 & 0 & 0 \\ \end{array} } \\ {\begin{array}{*{20}c} 1 & 0 & 0 \\ \end{array} } \\ {\begin{array}{*{20}c} {\begin{array}{*{20}c} 0 \\ 0 \\ \end{array} } & {\begin{array}{*{20}c} 1 \\ 0 \\ \end{array} } & {\begin{array}{*{20}c} 0 \\ 1 \\ \end{array} } \\ \end{array} } \\ \end{array} } \\ \end{array} } \right]\quad {\text{and}}\quad {\mathbf{d}} = \left[ {\begin{array}{*{20}l} k   \\ 0   \\ 0   \\ 0   \\ 0   \\ 0   \\ 0   \\ \end{array} } \right]$$

Then, the yield criterion can be expressed as a quadratic cone

42 $$\rho_{1} \ge \sqrt {\mathop \sum \limits_{j = 2}^{7} \rho_{j}^{2} }$$

given that

43 $$\rho_{1} = - \alpha I_{1} + k,\quad \sqrt {\mathop \sum \limits_{j = 2}^{7} \rho_{j}^{2} } = \sqrt{J_{2}}$$

Thus problem (37) can be rewritten as

44 $$\begin{gathered} \mathop {\min }\limits_{{({\hat{\varvec{\sigma }}},{\hat{\mathbf{r}}}{)}_{{n{ + 1}}} ,s_{1} ,s_{2} ,{{\varvec{\upxi}}}_{\sigma }^{{}} ,{{\varvec{\upxi}}}_{r}^{{}} ,{{\varvec{\uprho}}}^{i} }} \,\quad s_{1} + s_{2}   \\ {\text{subject to }}\quad {\mathbf{B}}^{{\text{T}}} {\hat{\varvec{\sigma }}}_{{n{ + 1}}} + {\mathbf{A}}^{{\text{T}}} {\hat{\mathbf{r}}}_{{n{ + 1}}} = {\tilde{\mathbf{f}}}   \\ \quad \quad \quad \quad \quad \quad \quad \;\;\;{{\varvec{\upxi}}}_{\sigma }^{{}} = \sqrt {\mathbf{C}} \Delta {\hat{\varvec{\sigma }}};\quad \;\;\;{{\varvec{\upxi}}}_{\sigma }^{{\text{T}}} {{\varvec{\upxi}}}_{\sigma }^{{}} \le 2s_{1}   \\ \quad \quad \quad\quad\quad \quad \quad \quad \;\;\;{{\varvec{\upxi}}}_{r}^{{}} = \Delta t\sqrt {\mathbf{D}} {\hat{\mathbf{r}}}_{{n{ + 1}}} ;\;\;\;{{\varvec{\upxi}}}_{r}^{{\text{T}}} {{\varvec{\upxi}}}_{r}^{{}} \le 2s_{2}   \\ \,\quad\quad\quad {{\varvec{\uprho}}}^{i} = {\mathbf{H}}{{\varvec{\sigma}}}_{{n{ + 1}}}^{i} + {\mathbf{d}}\;\;   \\ \,\quad\quad\quad\quad\quad\quad\quad\quad\quad\quad \rho_{1}^{i} \ge \sqrt {\mathop \sum \limits_{j = 2}^{7} (\rho_{j}^{i} )^{2} } ,\;\;i = 1,\;2,\;...,\;N_{{\text{G}}}   \\ \end{gathered}$$

which is a standard SOCP problem (32) and solved using the interior-point method [1] available in the modern optimization engine MOSEK [2]. An example of its detailed implementation with the solver MOSEK can be found in [53]. The non-associated plastic flow can be considered in the proposed computational framework following [32], where an associated computational plasticity scheme for non-associated frictional materials was developed. Specifically, the plastic potential function used in this study is

45 $$G = \alpha ^{\prime}I_{1} + \sqrt {J_{2} } - k^{\prime}$$

where *α*′ and *k*′ are the same expression as in Eq. (39) with the friction angle being replaced by the dilation angle *ψ*. To enforce the non-associated plastic flow rule, we approximate the yield function as

46 $$F \approx F^{*} = \alpha ^{\prime}I_{1} + \sqrt {J_{2} } - \tilde{k}$$

where $\tilde{k} = k - (\alpha - \alpha ^{\prime})I_{1}^{n - 1}$ is updated by the first invariant of the stress $I_{1}^{n - 1}$ obtained at the last time step. As such, we have $\partial F^{*} /\partial {\varvec{\sigma}} = \partial G/\partial {\varvec{\sigma}}$ which guarantees the associated computational scheme. The matrix **H** and **d** should also be modified as

47 $${\mathbf{H}} = \left[ {\begin{array}{*{20}c} { - \alpha ^{\prime}} & { - \alpha ^{\prime}} & { - \alpha ^{\prime}} & {\begin{array}{*{20}c} 0 & 0 & 0 \\ \end{array} } \\ {1/\sqrt 6 } & -{1/\sqrt 6 } & 0 & {\begin{array}{*{20}c} 0 & 0 & 0 \\ \end{array} } \\ 0 & {1/\sqrt 6 } & { - 1/\sqrt 6 } & {\begin{array}{*{20}c} 0 & 0 & 0 \\ \end{array} } \\ {\begin{array}{*{20}c} { - 1/\sqrt 6 } \\ 0 \\ {\begin{array}{*{20}c} 0 \\ 0 \\ \end{array} } \\ \end{array} } & {\begin{array}{*{20}c} 0 \\ 0 \\ {\begin{array}{*{20}c} 0 \\ 0 \\ \end{array} } \\ \end{array} } & {\begin{array}{*{20}c} {1/\sqrt 6 } \\ 0 \\ {\begin{array}{*{20}c} 0 \\ 0 \\ \end{array} } \\ \end{array} } & {\begin{array}{*{20}c} {\begin{array}{*{20}c} 0 & 0 & 0 \\ \end{array} } \\ {\begin{array}{*{20}c} 1 & 0 & 0 \\ \end{array} } \\ {\begin{array}{*{20}c} {\begin{array}{*{20}c} 0 \\ 0 \\ \end{array} } & {\begin{array}{*{20}c} 1 \\ 0 \\ \end{array} } & {\begin{array}{*{20}c} 0 \\ 1 \\ \end{array} } \\ \end{array} } \\ \end{array} } \\ \end{array} } \right]\quad {\text{and}}\;{\mathbf{d}} = \left[ {\begin{array}{*{20}l} {\tilde{k}}   \\ 0   \\ 0   \\ 0   \\ 0   \\ 0   \\ 0   \\ \end{array} } \right]$$

This scheme for handling non-associated plastic flow rule has been applied successfully to geotechnical problems including, but are not limited to, bearing capacity of foundations [32], slope stability [54], granular flow [67, 73], landslides [53, 65] and consolidation [68].

## Particle finite element technique

The particle finite element method (PFEM) combines the particle approach and the Lagrangian FEM method. In each time step of the PFEM calculation, the alpha-shape method [16] is used to identify the boundaries of the computational domain followed by mesh generation and Lagrangian finite element analysis. By doing so, the PFEM inherits the solid mathematical foundation of the FEM and the flexibility of particle approaches in modelling large deformation problems. A recent review of the development and application of the PFEM technique can be found in [13].

Given the potential of the PFEM for investigating many practical engineering applications, the explicit 3D PFEM has been developed for solving both fluid [18, 20] and solid problems [72]. Here, we extend the PFEM formulation developed in our previous study [64] to 3D modelling. The proposed 3D PFEM model inherits the advantages of the SOCP-FEM [4, 22, 33]. Its implicit time integration feature also enables the use of a relatively large time step which is required for quasi-static and low-to-medium dynamic geotechnical problems. The PFEM calculation within each time step includes: (i) updating the mesh nodes through the obtained incremental displacement in the last time step; (ii) identifying the boundaries of the computational domain using the alpha-shape method; (iii) generating a new mesh conforming to the identified boundaries; (iv) mapping field variables from the old mesh to the new mesh using the Unique Element Method [26]: for each new node, the old mesh containing this node is first determined and the values at the new node are interpolated based on the shape functions and the node values of the old mesh; (v) solving the equations over the new mesh by the Lagrangian FEM.

**Fig. 2:**
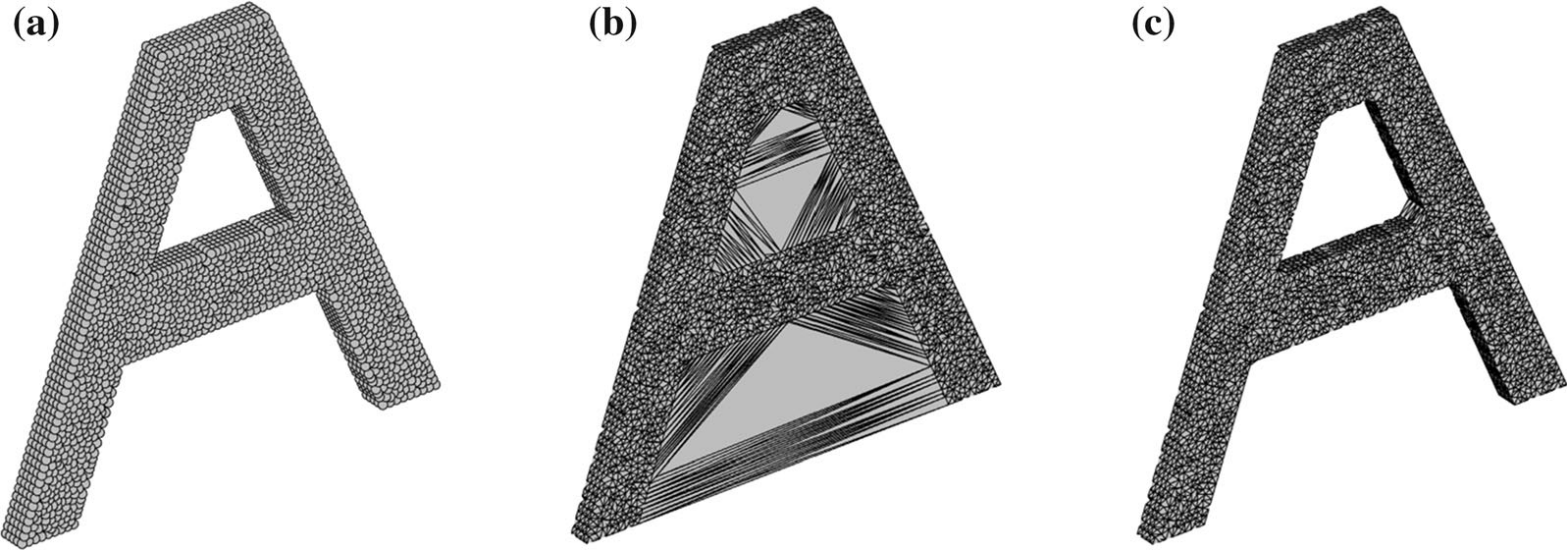
Boundary recognition using the alpha-shape method: **a** cloud of points; **b** Delaunay triangulation; **c** tetrahedral mesh after deleting tetrahedrons with the diameter of their circumscribed spheres larger than *αh*_*e*_ (*α* is a factor and *h*_*e*_ is the characteristic length of the mesh)

The alpha-shape method is implemented as follows. For a cloud of particles shown in Fig. 2a, 3D Delaunay triangulation is first used to construct a convex domain as illustrated in Fig. 2b. The diameter of each circumscribed sphere is calculated and compared to *αh*_*e*_, where α is a factor (1.2–1.6) [19, 64] and *h*_*e*_ is the characteristic length of the tetrahedral mesh which could be the average diameter of all circumscribed spheres. Elements with diameters great than *αh*_*e*_ are deleted; otherwise, they are retained. After this procedure, the boundaries are recognised. As in the 2D PFEM [64], finite element meshes of the 3D PFEM are also available when the boundary is identified (Fig. 2c). However, the quality of the meshes generated from this procedure can be low. Particularly, sliver elements may appear when the four nodes of a tetrahedral element are located nearly on a plane [12, 29]. These elements are likely to be distorted severely within a single time step when soil flows, which would consequently affect the simulation results producing unphysical solutions. To eliminate this issue, a smoothing technique is developed such that integration is carried out on cells/patches rather than finite element meshes [39, 62, 72, 74, 76]. To ensure mesh quality, in this study, the mesh topology and mesh nodes inside the domain are erased, and a remeshing operation of the identified domain is conducted using the robust mesh generator TetGen [23].

It should be stressed that the present PFEM technique is based on the infinitesimal strain assumption with an updated geometry, which is an approximation for large deformation analysis. Despite somewhat errors may be induced particularly for rotations, this modelling strategy has proven to be efficient in handling large deformation problems. The so-called sequential limit analysis is a typical example that was initially used for frame structure analysis with large displacements [61]. It was later extended to plane-strain problems of the von Mises model with non-linear isotropic hardening, and its convergence and validation were demonstrated [36]. In geomechanics, this method was used to analyse pipe-soil interactions during large amplitude cyclic lateral displacements where satisfactory agreements were achieved between the simulation results and laboratory testing data [30]. Additionally, the incremental small-deformation theory for large deformation analysis is also the core of the RITSS (Remeshing and Interpolation Technique with Small Strain) method which has been widely examined and used to analyse large-deformation geotechnical problems [26, 49, 52, 59]. It is also notable that this technique has been implemented in 2D PFEM to reproduce challenging large deformation problems such as water dam break, collapse of frictional materials and submarine-landslide-generated waves, for which satisfactory agreements between numerical simulations and laboratory results are achieved [69].

## Numerical examples

### Bending of an elastic cantilever beam

To verify our model for solving large deformation problems in 3D, we first simulate the classical solid mechanics problem of the bending of a thin elastic cantilever beam subjected to (i) a linear load and (ii) a moment. The first case is a problem of large deflection and moderate rotation while the second is of large rotation. The analytical solutions for both cases are available.

#### Bending under a linear load

**Fig. 3:**
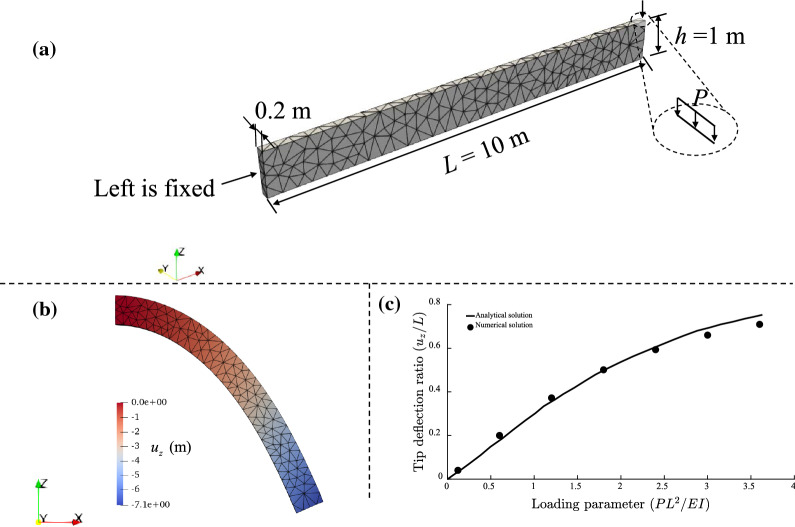
Bending of an elastic beam (with the Young’s modulus *E* = 1 GPa and Poisson’s ratio *υ* = 0.0) under a linear load *P*: **a** model configuration; **b** displacement in the *z* direction *u*_*z*_ under the maximum load of *P* = 3000 kPa; **c** comparison between our numerical simulation and the analytical solution for capturing the tip deflection ratio *u*_*z*_/*L* as a function of the loading parameter *PL*^2^/*EI*, where *I* is the inertial moment of the beam

In this example, a beam is subjected to a downward uniform linear load *P* as shown in Fig. 3a, which is a classical benchmark for large deformation analyses [37, 77]. The beam has a length of 10 m (in the *x* direction), a thickness of 0.2 m (in the *y* direction) and a height of 1 m (in the *z* direction). The Young’s modulus and Poisson’s ratio are *E* = 1 GPa and *υ* = 0.0, respectively, which are in line with those from [77]. The uniform load of *P* = 3000 kPa is applied along the top line of the right end of the beam in a slowly ramped manner to mimic a quasi-static condition. The beam is discretised using 693 elements (Fig. 3a). At the end of each time step, the geometry of the beam is updated based on the solved incremental displacement. The contour of the displacement in *z*-direction for *P* = 3000 kPa is plotted in Fig. 3b, which agrees well with the one in [77]. In Fig. 3c, we also show the tip deflection ratio *u*_*z*_/*L* as a function of the loading parameter *PL*^2^/*EI,* where *I* is the inertial moment of the beam. Numerical solutions are calculated with *P* = 100, 500, 1000, 1500, 2000, 2500 and 3000 kPa. Our simulation results show a good agreement with the analytical solution (Fig. 3c).

**Fig. 4:**
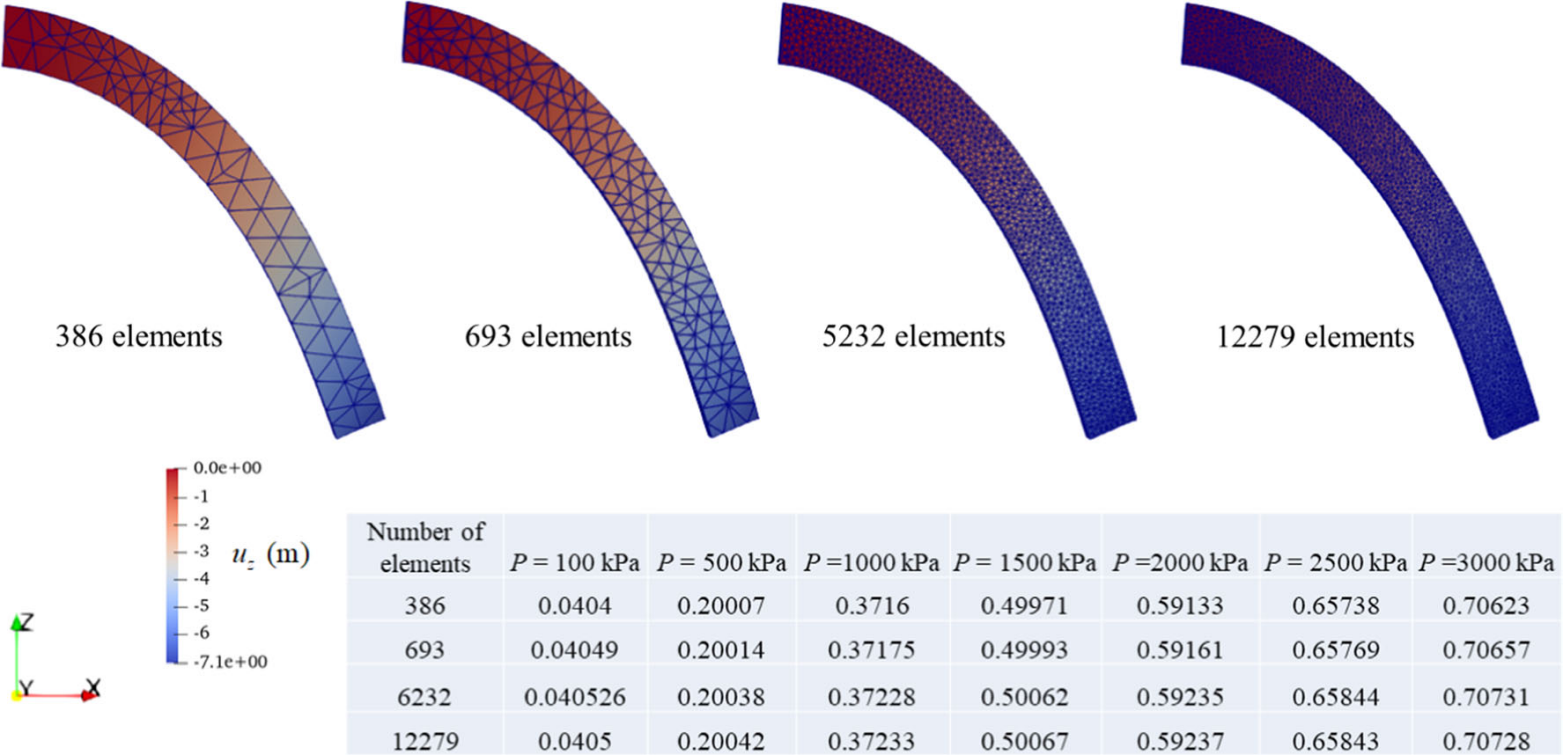
Final deformation of the beam under the maximum load of *P* = 3000 kPa with four mesh configurations and the calculated tip deflection ratios for several loading conditions. The contours illustrate the displacement *u*_*z*_ distribution

To investigate the convergence of the numerical solution, we also simulate this problem with three additional mesh configurations (386, 5232 and 12,279 elements). We show the final deformation of the beam under the maximum load of *P* = 3000 kPa and present the calculated tip deflection ratios obtained from the considered four mesh configurations under loads in Fig. 4. As can be seen, the deformations are nearly the same and the differences between tip deflection ratios are minor, which demonstrates the mesh convergence of the developed model.

#### Bending under a moment

**Fig. 5:**
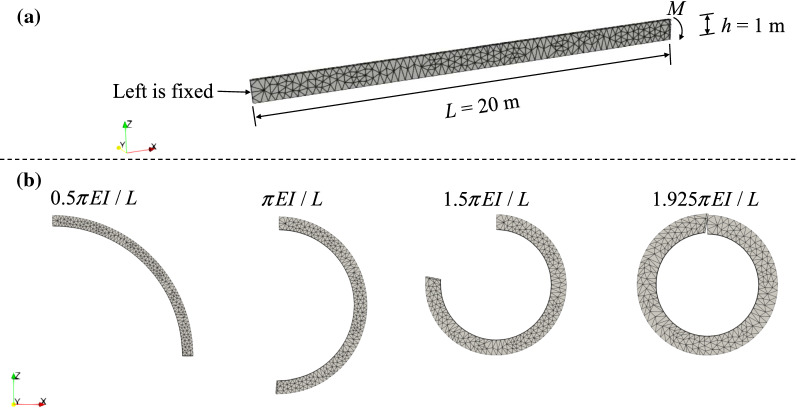
Bending of an elastic beam under a moment applied at the right end (with the Young’s modulus *E* = 5 GPa and Poisson’s ratio υ = 0.0): **a** model configuration; **b** modelled beam geometry at four instants

In this example, a beam is subjected to a moment at the right end, as shown in Fig. 5a. We model a longer beam with the length *L* being 20 m and the Young’s modulus *E* is 5 GPa. Other parameters are the same as those in the previous example. The beam is discretised using 1308 elements. According to [44, 77], the beam can be bent into a circle when the moment is *M* = 2π*EI*/*L*. In our simulation, the moment at the right end of the beam increases gradually from zero to 2π*EI*/*L*. The numerical results at four instants are displayed in Fig. 5b, where the beam is progressively bent into a nearly perfect circle with a small error of 3.75% compared to the analytical solution.

**Fig. 6:**
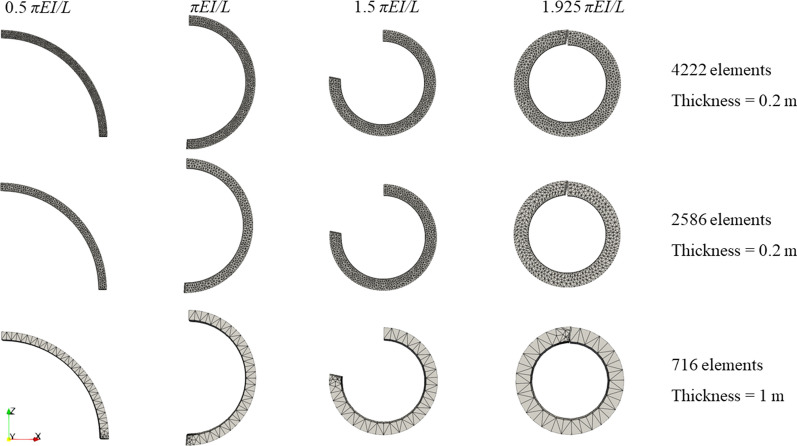
Modelled beam geometry at four instants with three more mesh configurations

Moreover, numerical results of three mesh configurations, i.e. two denser mesh configurations (4222 and 2586 elements) and a coarse mesh (716 elements) with thickness (along the y direction) being 1 m, are also shown in Fig. 6. Notably, no significant differences can be observed.

The above two examples (bending under a linear load and a moment) verify our model for simulating solid mechanics problems of large deflection and rotation.

### Solid collapse

**Fig. 7:**
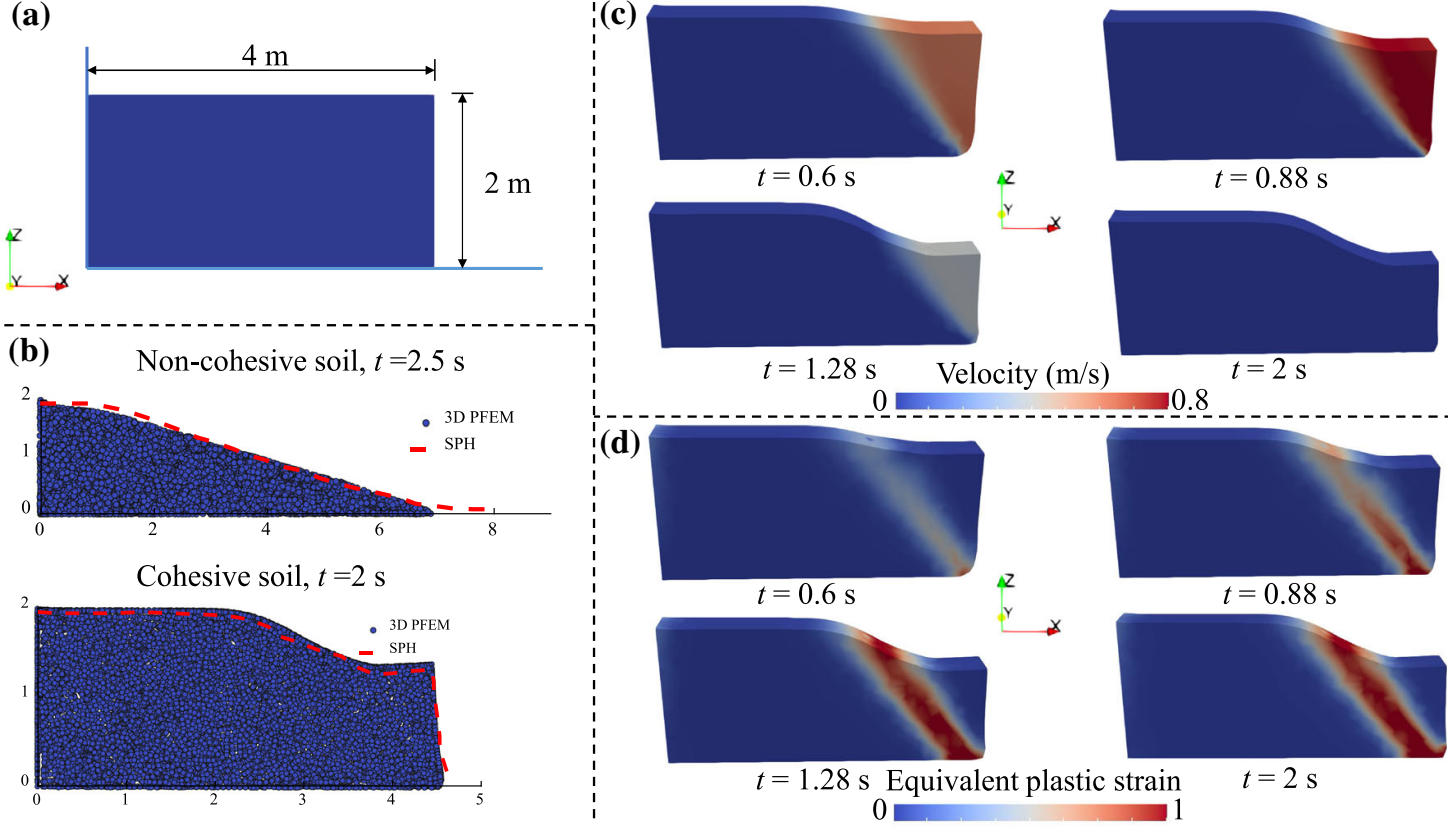
Collapse of a soil column: **a** model configuration; **b** final deposit profiles; **c** four instants showing the progressive failure of cohesive soils with the velocity distribution illustrated; **d** four instants showing the progressive failure of cohesive soils with the equivalent plastic strain distribution illustrated

The collapse of cohesive and non-cohesive soils driven by gravity is studied in this section. The soil column has a length of 4 m and a height of 2 m (Fig. 7a). Such problems have been simulated in the literature using the two-dimensional SPH method with the Drucker-Prager model under a plane strain condition (see [5, 11]). Here, we test our 3D PFEM model using the following examples. The material parameters are defined as follows [11]: the Young’s modulus *E* = 1.8 MPa, the Poisson’s ratio *υ* = 0.3, the density *ρ* = 1850 kg/m^3^, the friction angle *φ* = 25°, the dilation angle *ψ* = 0°, and the cohesion is *c* = 5 kPa for the cohesive case and zero for the non-cohesive case. The thickness of the soil column is set as 0.5 m in our 3D model. The domain is discretised using 4973 tetrahedral elements. The left and bottom surfaces are fixed while the front and back surfaces (normal to the *y* axis) are fixed in the *y* direction. A constant time step ∆*t* = 0.02 s is used in our simulation which is in contrast to a smaller time step ∆*t* = 1.5 × 10^–5^ s used in [11] where an explicit time integration scheme is adopted.

The final deposit profiles of the two cases calculated from our 3D PFEM modelling are shown in Fig. 7b, which are also compared to the 2D SPH simulation results from [11]. In general, a good agreement is obtained, which verifies the correctness of the 3D PFEM developed in our work. We also show the snapshots of the progressive failure of the cohesive soil at four instants with the contours of the velocity and equivalent plastic strain fields plotted in Fig. 7c and d, which also agree with those presented in [11].

**Fig. 8:**
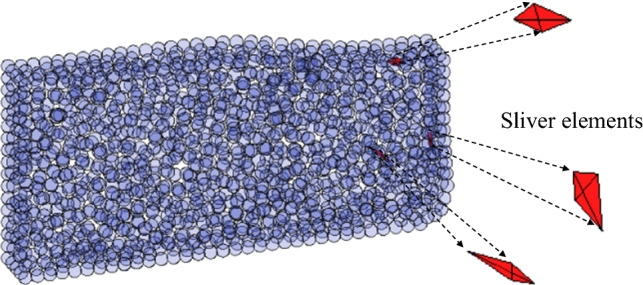
Sliver elements (marked in red) generated during the simulation without the remeshing procedure

To illustrate the necessity of remeshing after identifying the computational domain, we also analyse the problem using the meshes that are the by-product of the alpha-shape procedure of boundary identification. It is shown that sliver elements appear after several time steps of calculation (see Fig. 8).

**Fig. 9:**
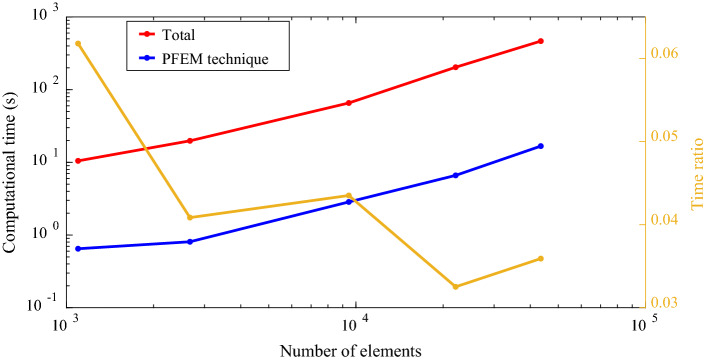
Computational time for one-step PFEM analysis using different meshes

The computational efficiency of our 3D PFEM model is also examined. We show the computational time for one time step of incremental analysis of the above example using different mesh configurations (with the number of elements: 1097, 2671, 9448, 22,097, and 43,499). The simulations are performed on a Dell desktop (i7-9700 CPU, 16 GB RAM). As seen in *Fig. **8**,* the computation time for the one-step simulation increases from 10 to 465 s when the number of elements increases from 1097 to 43,499. The computational time for re-identifying and remeshing the computational domain (PFEM technique) only occupies around 5% of the total computational time (*Fig. **9*).

### Soil slope failure

In practical geotechnical engineering applications, very few soil slope failure problems can be simplified as 2D systems. Actually, the geometry of a soil slope is usually very complex such that a full 3D direct simulation is needed. In this section, a 3D soil slope with a turning corner following the one used in [8, 42] is simulated using our 3D PFEM model.

**Fig. 10:**
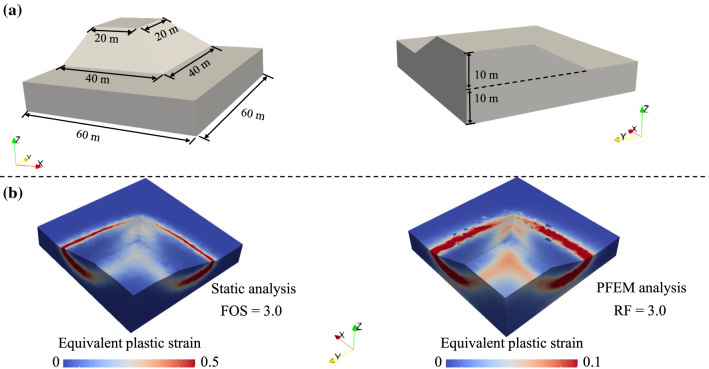
3D modelling of a soil slope failure: **a** model geometry; **b** identified slip surfaces through a static analysis and the PFEM dynamic simulation with reduction factor RF = 3

The geometry of the slope is shown in Fig. 10a. The slope has a height of 10 m and a slope ratio of 1:2. The material parameters include: the density *ρ* = 2000 kg/m^3^, the Young’s modulus *E* = 100 MPa, the Poisson’s ratio *υ* = 0.3, the cohesion *c* = 40 kPa, the friction angle *φ* = 20°, and the dilation angle *ψ* = 0°. We first perform a static stability analysis of the slope using the classical shear strength reduction method with a non-associated Drucker-Prager yield criterion:

48 $$c^{^{\prime}} = c/{\text{RF}}; \, \tan \varphi^{^{\prime}} = \tan \varphi /{\text{RF}}$$

where the superscripts of prime denote the reduced parameters in searching for the factor of safety (FOS) during iterations, and RF is the reduction factor (the ratio of the original parameters to the reduced parameters). We use the bisection method to approach the critical situation (RF = FOS), which is defined as the minimum strength parameters required to stabilise the slope [53]. In our simulation, the computational domain is discretised using 26,395 tetrahedral elements, and the model boundaries are set as (i) fixed for the bottom, (ii) roller for the lateral, and (iii) free for the top. The FOS calculated from our simulation is 3.0 which is between the calculated FOSs of 2.86 in [42] and 3.12 in [8]. The slip surface captured in the static analysis is shown in *Fig. **10**b*, which is illustrated by the field of equivalent plastic strain showing a good agreement with the simulation results in [8, 42]. To further present the capability of our 3D PFEM model in describing the entire pre- and post-failure processes of the slope, we re-analyse this problem using different reduced strength parameters ($c^{\prime}$ and $\varphi ^{\prime}$). We first consider the critical condition (RF = FOS) to study the dynamic deformation of the slope using the strength parameters reduced by the FOS calculated from the static analysis. The field of equivalent plastic strain of the final slope profile is plotted in Fig. 10b. It is shown that the slip surface (concentration of equivalent plastic strain) is consistent with the one from the static analysis. Comparing the final profile to the original slope geometry, it is observed that the slope geometry is nearly the same. This simulation indicates that the failure of homogeneous slope moves along the slip surface (which is the same as the one obtained from the static analysis) and further stabilises after a very limited deformation under critical condition, which is in line with our previous study on a two-dimensional homogeneous slope [53].

**Fig. 11:**
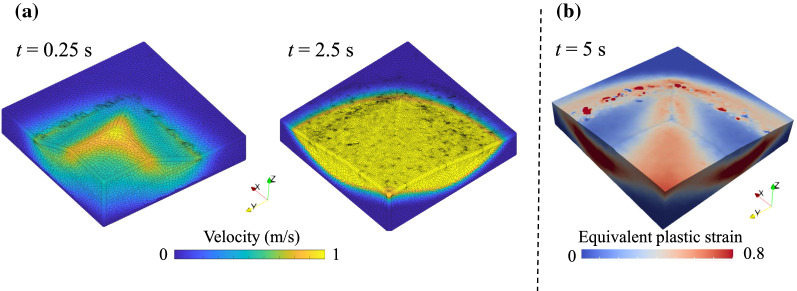
PFEM simulation results for the slope failure with the reduction factor RF = 5: **a** velocity distribution at two instants; **b** final failure pattern characterised by the distribution of equivalent plastic strain

To further present its capability in capturing rapid movement of soil flow, we then analyse the scenario of a larger reduction factor RF = 5 based on the PFEM model. To investigate its performance, we calculate the statistics of the mesh generated during the remeshing procedure using a built-in function of TetGen [23]. The data show that the largest and smallest dihedral angles are around 170° and 4°, respectively (sliver elements have dihedral angles close to 0° or 180° [23]). The number of elements with small dihedral angles (< 5°) is quite small (e.g. around 5), and the computational time for searching numerical solutions at each time step is almost the same, which implies the mesh quality is maintained good by TetGen [23]. The post-failure process in this case lasts for 5 s. The velocity distribution and the equivalent plastic strain are shown in Fig. 11, illustrating the slope failure process. As shown in Fig. 11a, the maximum velocity occurs at the top surface of the slope (see *t* = 0.25 s) and the failure rapidly propagates, producing a fan-shaped body at the end (see *t* = 2.5 s). Comparing the final failure pattern (Fig. 11b) to the slip surface in Fig. 10b, it is shown that the failed mass moves along the slip surface, while higher mobility (lower reduced parameters $c^{\prime}$ and $\varphi ^{\prime}$) causes a diffuse failure pattern. The simulation results suggest that the weakening effect of material is a key to unravelling the complexities of the dynamic process of slope and more realistic constitutive relationships are required (instead of the unphysical reduction factor used here).

## Conclusions

In this paper, a 3D PFEM model integrating elastoplasticity is developed for modelling soil flow problems of large deformation. In our PFEM model, the implicit finite element formulation with a mixed quadratic-linear tetrahedral element is developed based on a generalised Hellinger–Reissner variational principle. The final formulation is cast into a standard SOCP problem which can be resolved efficiently using the interior-point method. It is shown that the implicit feature enables the PFEM to use a relatively large time step, which is of great importance for solving geotechnical problems usually of either quasi-static or low-to-medium dynamic nature. The computational domain is constantly remeshed to avoid the generation of sliver elements. The proposed 3D PFEM for modelling large deformation solid mechanics problems is verified based on the classical beam deflection benchmark tests that demonstrated the accuracy of our model in capturing both large displacement and large rotation. Additionally, the capability and robustness of our model for simulating soil flows are demonstrated by modelling the collapse of a soil column using both cohesive and non-cohesive scenarios as well as an application to simulating the failure of a 3D soil slope with both the pre- and post-failure processes realistically captured. The proposed 3D PFEM model can serve as a basis for developing more advanced geoscientific models for identifying potential slope failure and predicting its sequential post-failure processes.
